# Radiolabeled Liposomes for Nuclear Imaging Probes

**DOI:** 10.3390/molecules28093798

**Published:** 2023-04-28

**Authors:** Ho Ying Low, Chang-Tong Yang, Bin Xia, Tao He, Winnie Wing Chuen Lam, David Chee Eng Ng

**Affiliations:** 1 Department of Nuclear Medicine and Molecular Imaging, Radiological Sciences Division, Singapore General Hospital, Outram Road, Singapore 169608, Singapore; low.ho.ying@sgh.com.sg (H.Y.L.); winnie.lam.w.c@singhealth.com.sg (W.W.C.L.); david.ng.c.e@singhealth.com.sg (D.C.E.N.); 2 Duke-NUS Medical School, 8 College Road, Singapore 169857, Singapore; 3 School of Chemistry and Chemical Engineering, Hefei University of Technology, Hefei 230009, China; xiabin@mail.hfut.edu.cn (B.X.); taohe@hfut.edu.cn (T.H.)

**Keywords:** liposomes, nuclear imaging probes, radiolabeling, radiopharmaceutical, theranostics

## Abstract

Quantitative nuclear imaging techniques are in high demand for various disease diagnostics and cancer theranostics. The non-invasive imaging modality requires radiotracing through the radioactive decay emission of the radionuclide. Current preclinical and clinical radiotracers, so-called nuclear imaging probes, are radioisotope-labeled small molecules. Liposomal radiotracers have been rapidly developing as novel nuclear imaging probes. The physicochemical properties and structural characteristics of liposomes have been elucidated to address their long circulation and stability as radiopharmaceuticals. Various radiolabeling methods for synthesizing radionuclides onto liposomes and synthesis strategies have been summarized to render them biocompatible and enable specific targeting. Through a variety of radionuclide labeling methods, radiolabeled liposomes for use as nuclear imaging probes can be obtained for in vivo biodistribution and specific targeting studies. The advantages of radiolabeled liposomes including their use as potential clinical nuclear imaging probes have been highlighted. This review is a comprehensive overview of all recently published liposomal SPECT and PET imaging probes.

## 1. Introduction

In the 1960s, liposome was first discovered by a British hematologist, Alec D Bangham, and was claimed as a potential drug carrier in the 1970s [1]. Liposomes are artificial spherical lipid vesicles containing either a unilamellar or multilamellar phospholipid bilayer. Typically, the lipid bilayer membrane of a liposome is made up of three major components: glycerophospholipids, sphingomyelins, and cholesterols. The phospholipid components assemble and aggregate in a cluster spontaneously and naturally where the polar heads are facing outward and the hydrophobic tails are facing inward, eventually forming an enclosed amphiphilic system with an aqueous center. This organization is driven by physical properties of the phospholipid, in particular, interaction between the hydrophilic head group and the hydrophobic tail of phospholipids [2].

In the past few decades, liposomes have been vigorously studied and have been suggested to be a promising drug delivery system in various aspects, especially in oncology [2]. Concerning their versatile structure and unique physiochemical properties, liposomes are able to carry a broad spectrum of materials to the desired sites, including biological materials such as nucleic acids, proteins, and lipids, therapeutic agents, immunomodulators, and radionuclides. For instance, hydrophilic active materials may be entrapped in the aqueous core, while hydrophobic active materials may be incorporated into the phospholipid bilayer either in “free” form or with chelators. This technological approach has been proven to not only improve the therapeutic pharmacokinetic and pharmacodynamic profile of the drug, but also increase the therapeutic index through effective drug delivery to the site.

**Scheme 1:**
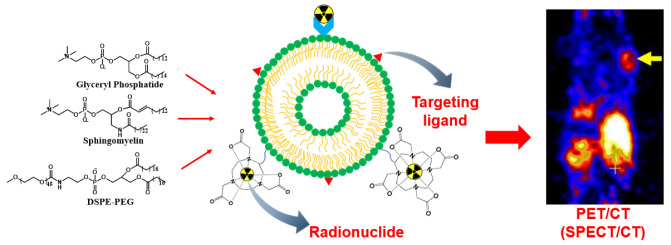
Typical liposome modifications for nuclear imaging.

**Scheme 2:**
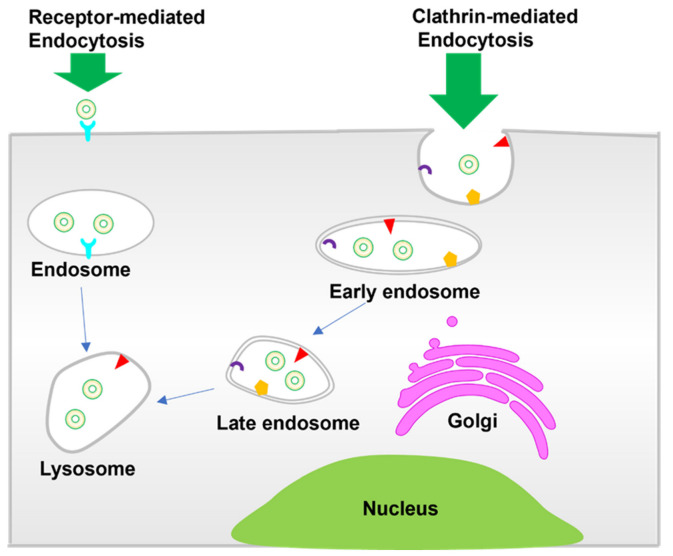
Cell internalization pathways of liposomes.

In order to expedite liposomes as effective drug carriers in clinical applications, it is fundamental to understand the structural and physiochemical properties of liposomes, since biological responses and behavior are mainly controlled by these parameters. Glyceryl phosphatide, sphingomyelin, and PEGylation liposomes, representing typical liposomes, have been widely applied in clinical use [3] (Scheme 1). These liposomes have been studied extensively as drug carriers or therapeutic agents. Scheme 2 shows the cell internalization pathways of liposomes. The endocytic route applies to most non-ligand-targeted liposomes for cell internalization, which relates to the size, shape, and surface charge of liposomes. Clathrin-mediated endocytosis plays an important role in the internalization of liposomes into cells [4]. Vesicle formation involves extensive protein machinery to create the spherical clathrin-coated pit in the membrane [5], which is further transported to early endosomes, lysosomes, or multivesicular bodies. Liposomes with a size of around 100 nm tend toward internalization via clathrin-mediated endocytosis [6,7]. In addition, receptor-mediated internalization of ligand-targeted liposomes has been widely developed to enhance cellular uptake. Folate receptor (FR), transferrin receptor (TfR), and epidermal growth factor receptor (EGFR), as typical examples, have been explored for active disease cell targeting [8]. The overexpressed FR on common tumor cells immensely promotes binding with folate-conjugated liposomes [9,10]. TfR is more highly expressed (100-fold higher) in tumor cells than normal cells, revealing itself an as attractive target for tumor theranostics through transferrin-conjugated liposomes [4,11].

Size and lipid composition are key factors that determine the circulation time of liposomes (half-life) in the system [12]. Liposomes with larger sizes show a relatively faster clearance rate than that of smaller liposomes as the larger surface membrane allows high serum protein absorption (so-called opsonization), and these serum proteins are recognized by cells of the mononuclear phagocytic system (MPS), leading to clearance in the liver and spleen in particular; liposomes larger than 200 nm are predominately removed by splenic macrophages, while liposomes that are smaller than 70 nm are mainly removed by liver Kupfer cells due to the limited pore size of liver sinusoidal filters [13]. On the contrary, SUVs with smaller surface membranes restrict surface absorption by the serum proteins, escaping recognition from the cells of the MPS and resulting in an extended circulation of liposomes in the blood; thus, SUVs are often preferred in studies.

Liposomes, as well as large molecules such as antibodies, can only accumulate in tumor sites either because tumor vasculature is leaky or due to the enhanced permeability and retention (EPR) effect [14]. However, high uptake by the MPS is observed. The mononuclear phagocytic system known as the reticuloendothelial system (RES) is modulated by macrophages and phagocytic cells in the liver, spleen, and bone marrow. Phagocytic cells catalyze liposomes by recognizing plasma and blood cells (e.g., complements or immunoglobulins) absorbed on the surface of liposomes. Hence, manipulation of liposomes is mandatory to evade the RES with the aim of prolonged circulatory longevity in the system, and prevention of the premature release of active materials increases the efficacy of delivery and the accumulation of active materials at desired sites [15]. Liposomes can accumulate at tumor sites when tumors have high endocytic activity. Such liposomes exhibit a short circulation time in the system due to rapid uptake by the MPS. Only a limited amount of active ingredients carried in liposomes can be distributed to targeted sites. The other disadvantage of this approach is that they tend to release active material before delivery to the target site, so-called pre-mature release. It is unstable during synthetic preparation because conventional liposomes are usually fabricated by natural phospholipids with unsaturated fatty acid groups that are prone to degradation [16]. All the above-mentioned obstacles have been found in in vivo studies; thus, it is necessary to develop new approach to overcome limitations.

Using surface-modified liposomes has proven an effective delivery method with high encapsulation efficiency and a long half-life for liposomes. Coating hydrophilic components such as polymer polyethylene glycol (PEG), silica acid, or monosialogangioside (GM1) on the surface of liposomes can limit the absorption of protein opsonins by liposomes and increase vascular permeability, which results in an enhanced accumulation of liposomes in tumors [2,17] owing to the increased hydrophilicity of liposome surfaces. These modified liposomes are also referred to as “long-circulating liposomes”. To obtain a long-circulating liposome, two parameters should always be considered, which are the size and lipid components of liposomes. Pegylated liposomal doxorubicin (DXR), also well known as Doxil^®^, is the first FDA-approved liposome formulation with a size of <100 nm subjected to multiple myeloma and ovarian cancer treatments. By using this pegylated liposomal formulation as a delivery vessel, significant improvement was noted in terms of pharmacological and therapeutic indexes [10]. Several liposomal formulations have been clinically approved, such as a daunorubicin-encapsulated liposome (DaunoXome^®^) for Kaposi’s sarcoma, a cytarabine-encapsulated liposome (DepoCyt^®^) for lymphomatous meningitis, and the recent vincristine-encapsulated liposome (Marqibo^®^) for acute lymphoblastic leukemia, and many more are still in preclinical or clinical studies [2,18,19]. Nonetheless, to achieve delivery with high specificity, liposomes need to be conjugated with functional group molecules such as proteins, antibodies, antibody fragments, carbohydrates, and other small molecules. It is crucial after surface modifications that liposomes are able to recognize cells that express corresponding receptors, and this mechanism is called active transport [20,21].

Herein, we first introduce the physicochemical properties and structural characteristics of liposomes and their effect on the duration of circulation as drug carriers. Various radiolabeling methods for liposomes involving radionuclides and synthesis strategies are then elucidated to render them biocompatible and capable of specific targeting. Lastly, a review is conducted of recent progress in the development of radiolabeled liposomes for use as nuclear imaging probes for current preclinical and potential clinical applications in nuclear medicine and their future aspects.

## 2. Why Radiolabeled Liposomes?

Bio-imaging techniques have been used for cancer theranostics to improve treatment outcomes and increase patient survival rates. The nuclear imaging techniques positron emission tomography (PET) and single photo emission computed tomography (SPECT) are the preferred modalities due to high sensitivity and deep tissue penetration. For nuclear imaging techniques, administration of a radiotracer is required for localizing and monitoring the tumors or lesions in a timely manner in preclinical and clinical practices [22]. Radiotracers, so-called nuclear imaging probes, contain radionuclides and vary with decay pathways and times, emission energies, and penetration levels. The images acquired from imaging techniques allow for examination of physiological processes in the area of interest and the quantitative and semi-quantitative evaluation of metabolites.

SPECT and PET offer high sensitivity (in the pico-nano-molar range) based on their underlying physical and technical principles, such as efficient attenuation. Both imaging modalities have been used extensively for the visualization of metabolic and physiological processes. However, due to low spatial resolution, nuclear imaging modalities suffer from the restricted visualization of the anatomic structure of cells. Combined with other imaging techniques, such as computerized tomography (CT) or magnetic resonance imaging (MRI), these modalities offer anatomical information regarding the location, size, and structure of the tumor or lesion areas (Table 1) [23,24,25,26]. Multimodal imaging that integrates functional and anatomical strengths has developed tremendously in terms of achieving precise and accurate physiopathological evaluation, better treatment advice, and enhanced patient management [24,27,28,29,30,31,32].

In nuclear medicine, a radiopharmaceutical is the combination of a biologically relevant molecule and a radionuclide and is used for visualizing distribution in organs or infection tissues, with detection of metabolic activity achieved through the PET or SPECT techniques [33,34]. Ideally, a radiopharmaceutical that can be used for disease diagnoses such as tumor detection and specific targeting for theranostics is always the goal. ^99m^Tc is the most extensively used radionuclide for SPECT imaging among all the gamma-ray emitting radionuclides [25]. With its optimal half-life and low gamma energy of 140 keV, ^99m^Tc is designed for diagnostic detection of various diseases and injuries in the brain, bone, lungs, kidney, thyroid, and many other organs and tissues [35]. Additionally, ^99m^Tc can be eluted from a ^99^Mo/^99m^Tc generator that is commercially available and inexpensive. The ease of production benefits manufacturers, medical faculties, and patients. PET radionuclides such as ^18^F are often used in clinical PET imaging due to their optimal half-life that enables them to be distributed in the body, while radionuclides ^13^N and ^15^O are unfavored in clinical diagnostic imaging in oncology due to their shorter half-lives and the requirement for cyclotron for on-site radionuclide production. ^18^F-fludeoxyglucose (^18^F-FDG) has been widely used as a radioactive tracer in routine clinical practice, and the mechanism underlying the accumulation of ^18^F-FDG in the tumor is related to the glucose metabolism of the cells; a high ^18^F-FDG concentration at the site indicates high glucose consumption, and in most cases, tumors have a high glucose metabolism [36,37].

Besides ^18^F-FDG, current clinical PET radiotracers are small molecules which exhibit a fast metabolism and non-specific distribution. The development of macromolecular nuclear imaging probes is needed to overcome the unfavorable characteristics of small molecular tracers. Nanoparticle-based imaging probes have shown advantages over the traditional small molecular imaging probes due to well-known strengths such as a large surface area that renders them biocompatible, allows for surface modifications for specific targeting, and even enables multimodal imaging probes to be obtained. Radiolabeled nanoparticles can be conjugated with various targeting molecules, such as antibodies and peptides, resulting in nuclear imaging probes for tumor-specific applications or probes with higher affinity to biomolecules or cells [38,39,40,41].

Liposomes are biocompatible and biodegradable vesicles that can be an ideal delivery platform for various radionuclides. Liposomes have drawn attention for drug delivery applications, particularly in oncology. Currently, the tumor targeting of most liposomes is due to the EPR effect, but the EPR effect is heterogeneous and depends on several parameters. Modifications of liposomes can be versatile (such as enhancement of circulation longevity) and increase entrapment efficiency, selectivity, and specificity without inducing toxicity or sacrificing stability. Nuclear imaging techniques are a group of non-invasive techniques that offer whole-body quantification and dynamic distribution of radiolabeled liposomes. Based on this, nuclear imaging is a powerful imaging modality for determining the pharmacokinetics and the tissular distribution of liposomes. Encapsulating radionuclides within the liposome is one of the approaches for the radiolabeling of liposomes. The external layers of the lipid membrane serve as a barrier, protecting the bioactive payload from enzyme breakdown, degradation, or protein binding [42,43,44]. Prior to the radiolabeling process, the choice of radionuclide and liposomal formulation should be taken into consideration with respect to the research purposes and the desired applications of the radioliposomes. The half-life of radionuclides should be compatible with the biological half-life of the liposomes. A large variety of liposomal formulations can be obtained by engineering the characteristics of liposomes. For instance, ^67^Ga-citrate is not an ideal radiotracer for abdominal imaging since the excretion pathway is through colon, resulting in false-positive outcomes in the abdominal areas [45]. Thus, new strategies for utilizing well-described liposomes as carriers have been proposed to optimize the application of radiopharmaceuticals at the clinical level.

On the other hand, the development of pretargeting strategies for liposomes in nuclear imaging is promising. In vivo pretargeting approaches have been employed for nuclear imaging and therapy through the injection of a targeting agent, such as tumor-targeting antibodies, followed by the injection of a radioisotope that binds to the targeting agent. These two components combine and accumulate at the target, exhibiting favorable pharmacokinetics. Decoupling of the targeting agent and radioisotope in pretargeting strategies reduces uptake of the radioisotope in non-target tissues [46,47,48]. 

Table 2 summarizes the radiolabeling methods of liposomes, their advantages, and their disadvantages. A suitable radiolabeling method that is constructed for certain radionuclide-labeled liposome formulations should be determined according to the intended application and the selection of radionuclides and liposomes. Optimization of a radiolabeling method is critical since the method design directly influences the quality of radioliposomes with respect to encapsulation efficiency and physical stability. Radiolabeling methods have been established using passive loading techniques or post-loading techniques. Radionuclides with different chemical and physical properties can be either encapsulated into the inner aqueous core of liposomes or attached to the surface of liposomes with or without the assistance of chelators, which greatly impacts the stability of radioliposomes.

The passive encapsulation method was the first radiolabeling method established by McDougall and co-workers. It simply depends on the hydrophilicity of radionuclides [42,49,50,51,52,53,59,85,86,87]. The radionuclide is incubated directly with a lipid solution during the liposome manufacturing process. Due to the hydrophilicity of intraliposomal space, the hydrophilic radionuclide is entrapped in the inner aqueous space. Since the passive encapsulation method failed to retain radionuclides within the liposome, a novel labeling method was constructed by adding a coordination chelator or other chelating agent to strengthen the structure of radiolabeled liposomes through covalent binding of the liposome and chelation with the radionuclide. This labeling method is capable of increasing the in vitro and in vivo stability of radionuclide-labeled liposomes. Identically, the radionuclide was encapsulated in the intraliposomal space during the radiolabeling process with the help of a chelating agent. It is important to note that knowledge of the coordination chemistry of each radionuclide and relevant chelator is required to accurately propose a suitable chelating agent [88,89].

A bifunctional chelating agent plays an important role in radiolabeling methods by ensuring a stable radioliposome complex, which is achieved by strengthening the binding between the radionuclide and preformed liposome. An advantage of using a bifunctional chelator is that labeling of a radionuclide to a liposome can be achieved after liposome preparation; thus, a faster and more efficient radiolabeling process can be carried out prior to administration [90]. By optimizing synthetic parameters, high labeling efficiency and in vitro and in vivo stability can be achieved for radionuclide-labeled liposomes. During liposome synthesis, a chelator is directly conjugated with a lipid membrane, forming a chelator–liposome complex that renders the chelator capable of allowing for chelation with the radionuclides. Generally used bifunctional chelators include *N*-hydroxy succinimidyl hydrazine nicotinate hydrochloride (HYNIC) for ^99m^Tc and ^111^In radiolabeling; tetraxetan, 2,2′,2′′,2′′′-(1,4,7,10-tetraazacyclododecane-1,4,7,10-tetrayl)tetraacetic acid (DOTA), for ^64^Cu and ^52^Mn radiolabeling; desferrioxamine (DFO) for ^89^Zr radiolabeling; diethylenetriaminepentaacetic acid (DTPA) for ^99m^Tc, ^111^In, and ^159^Gd radiolabeling; and DOTA for therapeutic radionuclide ^225^Ac [62,65,66,90,91,92,93,94,95,96,97]. In early studies, radiolabeling of ^99m^Tc and ^67^Ga for liposomes using DTPA was recorded at 95 ± 5% efficiency for both, though a high release of content in vitro (5% for ^67^Ga and 30% for ^99m^Tc after 2 h incubation) was noted, which could be problematic for administration; the high release of radionuclides was presumably due to enzymatic cleavage of radionuclides from liposomes or high exposure to blood serum components during circulation [66,98]. A very efficient labeling method using HYNIC as a chelating agent that conjugated to 1,2-distearoyl-sn-glycerol-3-posphoethanolamine (DSPE) on the surface of liposomes, which was proposed by Laverman et al., significantly increased in vitro stability and enhanced pharmacokinetic parameters [67]. In addition, a purification procedure was not required after the labeling process due to high labeling yield [69,99]. Recently, a study from Varga et al. used a novel labeling procedure to label ^99m^Tc-tricarbonyl [^99m^Tc(H_2_O)_3_(CO)_3_]^+^ onto liposomes that mimicked FDA-approved liposomal formulations Epaxal^®^ and Inflexal V^®^, with thiol groups subsequently formed after reaction with 2-iminothiolane (2-IT) [70,71,72]. Labeling efficiency in the range of 90–95% was obtained alongside better stability in vitro, with only 6% dissociation of ^99m^Tc from liposomes after 2 h incubation and prolonged radiostability for 108 min in vivo.

Owing to their physicochemical properties, ^68^Ga and ^64^Cu have advantages when trapped in the intraliposomal space stably via chelation. There is no established radiolabeling method for PET radionuclide-labeled liposomes. Recently, an efficient remote radiolabeling method constructed for PET radionuclides was reported [63,68]. Labeling of ^64^Cu to a liposome that contained hydroxyquinoline (HQ) as an ionophore and encapsulated DOTA as a chelator yielded more than 95% stable ^64^Cu-labeled liposomes in blood at 1 h of incubation and surprisingly high in vitro stability of >99% after 24 h of incubation. A biodistribution study showed high uptake of this radioliposome in tumors, suggesting its potential in diagnostic imaging and drug delivery, which was reported by Locke et al. [64].

Small molecules such as 8-hydroxyquinoline (8-HQ), acetylacetone, and tropolone have also been tested for the transport of radionuclides (^111^In, ^177^Lu, ^99m^Tc, and ^67^Ga) into the intraliposomal space through the use of nitrilotriacetic acid (NTA) for SPECT imaging [54,55,56,57,58]. A hydrophilic ion or an ionophore that is incorporated into the lipid membrane is involved in the radiolabeling process to facilitate the transportation of radionuclides across the lipid bilayer [86]. Hydrophilic ion transporters such as A23187 (calcimycin) are usually inserted into the lipid bilayer, forming a channel that allows for the passage of radionuclides, ^111^In specifically, into a liposome. The radionuclide ^111^In conjugated with NTA enters the inner core of the performed liposome [58]. Improved labeling efficiency (>90%) was reported even after the sonification process. It was elucidated that liposomes served as external shielding that protected the radionuclides from chemical degradation and reduced exposure to blood proteins.

Radiochemical yield and purity (%RCP) for the radiolabeling of liposomes has to be assessed. Radiolabeling can be characterized by high-performance liquid chromatography (HPLC) or thin-layer chromatography (TLC) using silica gel. For the HPLC technique, intact radiolabeled liposomes that are eluted are shown in radiochromatography, and integration of the radiometric signal represents the purity of the radiolabeled liposome [73]. For TLC, mobile phase is needed to separate the different particle sizes of liposomes on the silica gel, which is followed by measurement of radioactivity using a gamma counter [74,75]. In addition, radionuclide loading efficiency is an important parameter indicating the amount of active ingredients entrapped in the liposome. Low loading efficiency will lead to the leakage of active ingredients into the system, so-called “premature drug release”, which directly affects the efficacy of its in vivo delivery. The analysis of encapsulation efficiency can be determined using several technologies, such as a spectrophotometer, HPLC, or TLC [75].

## 3. Radiolabeled Liposomal Nuclear Imaging Probes

Table 3 summarizes all radiolabeled liposomal nuclear imaging probes with current, preclinical, and potential clinical applications. Liposomes labeled with ^99m^Tc, ^111^In, or ^67^Ga complexes were investigated extensively in different conditions. Various radiolabeled liposomes have been tested on different animal models at the preclinical stage, and the intention behind these studies is to understand their potential to act as clinical imaging probes for cancer, infections, atherosclerotic plaques, and blood pooling based on the biodistribution of radioliposomes [76,77,78,79,80,81,82]. In the meantime, as a novel drug delivery platform, various targeting moieties or agents can be loaded into liposomes to improve their biodistribution and therapeutic index at sites of interest. Most of the tested radioliposomes were labeled with SPECT radionuclides due to feasibility and favorable half-lives.

Although extensive efforts have been devoted to pharmacokinetic studies of radioliposomes, the research outcomes have been unsatisfactory in the last few decades due to various factors, such as rapid blood clearance, early drug release, low bioavailability, and non-targeted delivery. For example, ^99m^Tc-labeled liposomes failed to accumulate in several types of tumors even though similar tissue distributions of radioliposomes were found in these models (particularly high in the liver and spleen, moderate accumulation in the kidney and bone marrow) [75,86]. It was deduced that accumulation of ^99m^Tc-labeled liposomes is tumor-specific, and in vivo behavior of liposomes is strongly influenced by the characteristics of liposomes, test model selection, and targets [83]. Presant et al. demonstrated the potential of ^111^In-labeled liposomes as an imaging probe for cancer detection. The ^111^In-labeled liposome VesCan^®^ was the first liposome-based radiopharmaceutical reported for imaging of tumor metastasis with high specificity and sensitivity (70–80% sensitivity, >85% specificity) in animals. However, in clinical trials, the low detection sensitivity of VesCan^®^ for metastatic tumors led to failure in phase III trials, even though there were no reported side effects [84,100,101,102,103].

With developed technologies available in the field, the design of radioliposomes for particular aims can be established for satisfactory outcomes, facilitating the use of radioliposomes in preclinical and promising clinical applications. A study using ^99m^Tc-HMPAO-labeled liposomes was previously reported [85]. The incubation of HMPAO and ^99m^Tc allowed for the formation of a lipophilic complex prior to incubation with liposomes due to its high affinity for ^99m^Tc. This lipophilic ^99m^Tc complex can easily cross the lipid membrane, and once it enters intraliposomal space, the lipophilicity of the ^99m^Tc-chelate will be altered by chemical interaction with glutathione (GSH), leaving the ^99m^Tc-chelate stably trapped in intraliposomal space since the lipid membrane is impermeable to the hydrophilic molecules. Although a purification procedure is required for this method, the ^99m^Tc-HMPAO radioliposome was first reported to be stable in vivo with labeling efficiency ranging from 60% to 85% [86,104,105,106]. It has been found that the delivery efficiency of drug-encapsulated liposomes and their retention in tumor sites are closely related to the vascular permeability of tumors in terms of blood perfusion level and microvascular density [107]. In this study, the anti-tumor activity and in vivo biodistribution of ^111^In-encapsulated liposomes containing doxorubicin (Doxil^®^) was evaluated in human ovarian cancer mouse models, and the outcomes varied in four different cancer subtypes (Caov-3, SK-OV-3, KURAMOCHI, and TOV-112D). The anti-tumor activity of ^111^In-labeled Doxil^®^ was correlated to the accumulation of Doxil^®^ in various cancer subtypes. The Caov-2 cancer subtype showed the highest uptake of Doxil^®^ in the tumor, followed by SK-OV-3, while the uptake of Doxil^®^ in KURAMOCHI was the least. A significant reduction in tumor volume was only reported in Caov-3 and SK-OV-3 cancers after administration of Doxil^®^. Based on this finding, Ken Ito et al. suggested using an active targeting delivery approach for future experiments to ensure sufficiently high delivery efficiency and therapeutic effects in various tumor subtypes.

As a highly versatile lipid vessel, liposomes can carry multiple functional molecules or payloads, either by encapsulation or incorporation. By co-loading a therapeutic agent and a diagnostic agent into a single liposome, theranostics can be carried out at sites of interest while performing non-invasive in vivo visualization in real time. In this regard, the pharmacological effects of drug-encapsulated liposomes and the therapeutic efficacy of the respective drug formulation can be assessed after administration. This approach was reported by Monteiro and co-workers in their biodistribution study of paclitaxel (PTX), which was encapsulated in liposomes with several formulations; the PTX was loaded into a pH-sensitive, long-circulating liposome and demonstrated a significant improvement in in vivo distribution with reduced PTX toxicity [130]. Obstructions such as poor solubility, a low release of PTX, and low therapeutic efficacy were reported previously when PTX was loaded into conventional liposomes or other nanosystems. By loading PTX into pH-sensitive liposomes, the release of PTX can be easily modulated by pH, with PTX entrapped in the inner phase of liposomes stably at pH 7.4 or released from liposomes when pH is low. This approach not only reduces unnecessary retention in non-targeted organs or tissues but also increases the possibility of uptake by cells, therefore enhancing therapeutic indexes. High retention of PTX-encapsulated ^99m^Tc-labeled liposomes (^99m^Tc-SpHL-PTX and ^99m^Tc-SpHL-folate-PT) in tumors was reported at 4 h and 8 h post intravenous injection, and accumulations in RES organs such as the liver, spleen, and kidney were observed in both healthy and tumor-bearing mice [75]. The precise locations of tumors were shown in the clear scintigraphic images of PTX-encapsulated ^99m^Tc-liposomes with a significantly high tumor-to-muscle ratio, whereas a non-specific uptake of liposome-free PTX-^99m^T in the intestine was found in images of liposome-free PTX-^99m^Tc. This study has highlighted the importance of radioliposomes in diagnostic imaging due to their high delivery efficiency; however, further investigation of the therapeutic efficacy of this drug-encapsulated radioliposome should be carried out to demonstrate possibly enhanced theranostics.

**Figure 1:**
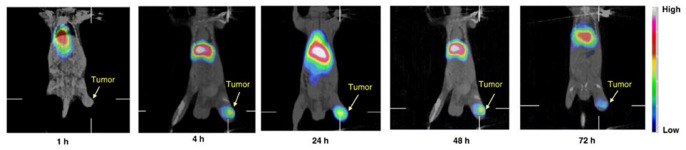
MicroSPECT/CT and WBAR images of ^188^Re-DXR-liposome targeting C26 tumors in BALB/c mice. ^188^Re-DXR-liposome, containing 22.2 MBq of ^188^Re, was administered to each mouse via intravenous injection. The microSPECT images were acquired at 1, 4, 24, 48, and 72 h after injection. The WBAR image was followed by microSPECT image acquisition at 72 h after injection of ^188^Re- DXR-liposome with the animal in the same position. Reprinted with permission from Ref. [114]. Copyright 2010 Elsevier.

Radionuclides such as ^111^In, ^67^Cu, ^188^Re, and ^131^I have been used as therapeutic agents in nuclear medicine. Among all of them, rhenium is a favorable radionuclide owing to its unique physical properties, which includes the release of both beta- and gamma-radiations at 2.12 MeV and 155 keV, respectively, allowing for cancer therapy and tracking [108,109]. Similar to ^99m^Tc, it can be routinely eluted from a ^188^W/^188^Re generator and made available for daily usage. A radiolabeling approach using the lipophilic chelator *N*,*N*-bis(2-mercaptoethyl)-*N*’,*N*’-diethyl-ethylenediamine (BMEDA) for ^188^Re and ^99m^Tc with a pH-sensitive liposome containing ammonium sulfate (pH 5.5) was reported by Bao and co-workers. Differences in the pH of the liposome exterior and interior were achieved through washing with PBS at pH 7.4, allowing the ^99m^Tc- or ^188^Re-BMEDA complex to enter the intraliposomal phase easily. The presence of ammonium sulfate causes BMEDA to be protonated and converted toward hydrophilicity, eventually trapping it in the inner aqueous core together with the radionuclide, while dissociated NH_3_ was free from the inner core of the liposome [60,61,104]. This approach has advantages for the labeling of radionuclides to drug-loaded liposomal formulations; thus, the resulting radioliposomes have potential in theranostic applications [110,111,112]. Numerous biodistribution and pharmacokinetic assessments of ^188^Re-BMEDA liposomes have been carried out in preclinical studies. These ^188^Re-labeled liposomes have demonstrated high uptake in tumors, the liver, and the spleen and enhanced therapeutic effects [113,114,115,116]. Chang et al. reported the use of ^188^Re-DXR liposomes in evaluation of the therapeutic effect of liposomal doxorubicin in tumor-bearing mice (Figure 1) [114]. An increased accumulation of ^188^Re-DXR-liposome in tumors was reported up to 48 h post intravenous injection, and tumor-to-muscle ratio was significantly higher (19 ± 5.7 after 24 h post administration). In addition, the tumor-bearing mice injected with ^188^Re-DXR-liposomes showed the highest survival rate, and tumor volume was drastically reduced. This preclinical outcome indicates the feasibility of using ^188^Re-DXR-liposomes as a theranostic agent. The same group later investigated ^188^Re-DXR-liposomes-BBN in Ar42J pancreatic tumor-bearing mice and a similar outcome was reported [115].

While radiolabeled liposomes for SPECT imaging are advantageous for nuclear medicine, the demand for quantitative PET imaging has grown fast recently, thus promoting the development of novel liposomal PET probes for theranostics. With their high sensitivity and quantitative images, positron-emitting nuclides such as ^18^F- and ^68^Ga-labeled liposomes have been investigated for their applications in in vivo biodistribution since the early 2000s [50,117,118]. A novel dual radionuclide labeling technique was reported in an in vivo biodistribution study [119]. In this study, [^18^F]-FDG was encapsulated into the intraliposomal space of [^111^In]-liposome, and in vivo biodistribution was characterized using SPECT/CT and PET/CT. This dual radionuclides–liposome technique requires two radionuclides emitting different emission energies, which subsequently enables the use of multimodality imaging techniques. The study of dual radionuclide-labeled liposomes enables images with crucial disease information to be generated from both modalities, promoting better data interpretation. Moreover, it can be used to assess the content of drug release from liposomes since the outcome is a correlation of the delivery efficiency of liposomes and the therapeutic efficiency of the drug-encapsulated liposomal formulation.

**Figure 2:**
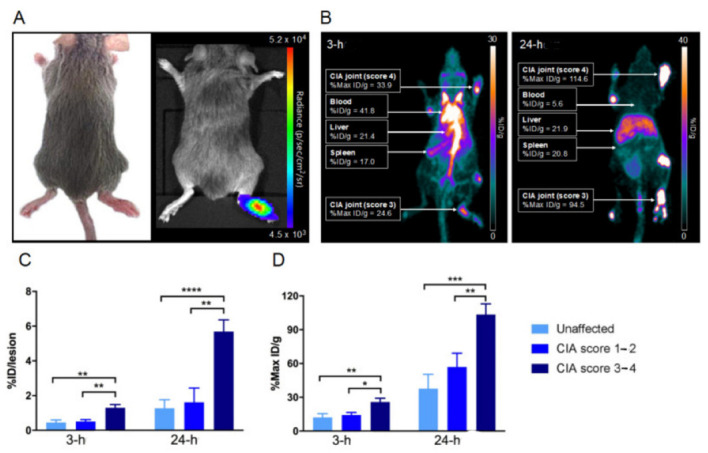
Collagen-induced arthritis in a DBA/1 mouse model using chicken type II collagen. (**A**) Representative images of (left) joints displaying arthritis 30 days after immunization, including swelling and erythema of the right tarsus, and (right) lucigenin BLI illustrating the inflammatory condition of the right tarsus on day 30 after immunization (same mouse). (**B**) Coronal whole-body distribution of ^64^Cu-liposomes in a CIA mouse. Three-hour and twenty-four-hour PET scans with liposomal activity (%Max ID/g) in the inflamed foci. Carpal and tarsal joints were clinically scored 4 and 3, respectively. Values for blood, liver, and spleen (%ID/g) radio-labeled liposomal activity after 3 h and 24 h are shown. (**C**) Mean joint accumulation (%ID/lesion ± SEM) and (**D**) maximum accumulation in the lesion (Max %ID/g ± SEM). After iv. injection of ^64^Cu-liposomes, 3 h and 24 h scans were performed, and results show significantly increased amounts of ^64^Cu-liposomes accumulated in joints with a high clinical score. Data are presented as mean ± SEM. Significant differences represented as * *p* < 0.05; ** *p* < 0.01; *** *p* < 0.001; **** *p* < 0.0001; paired t-test. Reprinted with permission from Ref. [127]. Copyright 2020 Elsevier.

**Figure 3:**
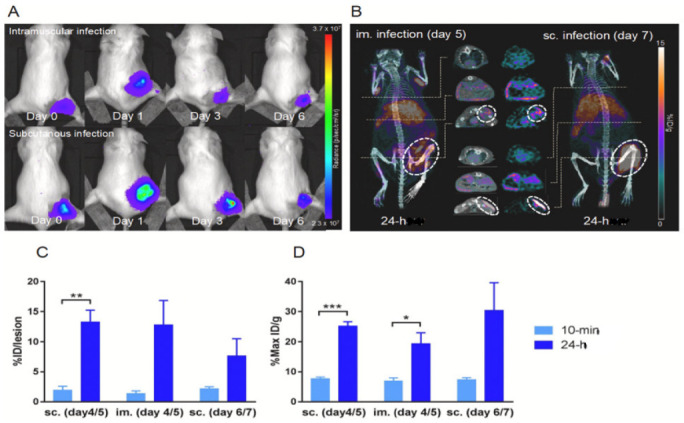
^64^Cu-loaded liposome PET/CT of a *S. aureus* Xen29 infectious mouse model. (**A**) BLI of female NMRI mice sc. or im. injected with *S. aureus* Xen29. Mice were scanned on day 0, 1, 3, and 6 after bacterial inoculation. (**B**) PET/CT images of *S. aureus* Xen29 infection 24 h after iv. injection with ^64^Cu-liposomes. White rings encircle lesion sites and ^64^Cu-liposome activity. (**C**,**D**) Activity levels of ^64^Cu-liposomes on PET/CT scans performed on day 4 and 5 (acute) and on day 6 and 7 (chronic) after bacterial inoculation. All mice were scanned 10 min and 24 h after injection with ^64^Cu-liposomes. Data are presented as mean ± SEM. Significant differences presented as * *p* < 0.05; ** *p* < 0.01; *** *p* < 0.001; paired *t*-test. sc.: subcutaneous; im.: intramuscular. Reprinted with permission from Ref. [127]. Copyright 2020 Elsevier.

^18^F-FDG- and ^68^Ga-labeled liposomes were used in early studies, but applications are often restricted by associated roadblocks; for example, their natural metabolic pathway could be confused in data interpretation, their short half-lives make them difficult to use in the latter portion of tumor staging, and readministration of PET tracers is necessary in some cases [117,120]. Therefore, new approaches using radionuclides such as ^64^Cu (t_1/2_, 12.7 h) and ^89^Zr (t_1/2_, 78.4 h) to label liposomes have emerged. With their longer half-lives, these radiolabeled liposomes are suitable as imaging probes for theranostics. In this regard, ^64^Cu is a radionuclide suitable for the labeling of liposomes, resulting in a longer tracking period of up to 48 h. Histological evidence has proven that ^64^Cu is able to detect malignant tumors effectively in various organs, which makes ^64^Cu preferred for tumor detection and staging [131]. Chelators that have been used to chelate with ^64^Cu include DOTA, BAT, TETA-PDP, DSPE, DPPE, and DPPC [74,121,131]. Several studies of ^64^Cu-labeled liposomes mainly focused on their biodistribution for clinical practice [122,123,124,125,126,127,132]. A few points can be drawn based on these studies of ^64^Cu-liposomes: First, ^64^Cu-labeled liposomes are stable in both in vitro and in vivo tests. Second, high uptake in tumors enables precise localization of tumors in images acquired by PET. Third, early tumor (neoplasm) detection can be achieved due to the high sensitivity of ^64^Cu-liposomes to cell proliferation, thereby improving the chances of survival for patients [131]. Besides cancer detection, ^64^Cu-labeled liposomes have successfully been applied for inflammation and infection lesion localization. The inflamed joints (Figure 2B) and areas infected by *Staphylococcus aureus* (Figure 3B) can be visualized on PET images, with an elevated accumulation of ^64^Cu-labeled liposomes over time observed after administration of ^64^Cu-liposomes [127]. In addition, the amount of ^64^Cu-liposomes was directly reflected in the collagen-induced arthritis (CIA) score; the higher the CIA scores, the higher the accumulation of ^64^Cu-labeled liposomes found at the sites (Figure 2C,D). A gradually reduced bioluminescent signal at the lesions was noticeable on day 6 (Figure 3A,C,D), indicating the theranostic potential of ^64^Cu-labeled liposomes for inflammation and infection lesions.

^89^Zr has a longer half-life radionuclide than ^64^Cu and is an emerging PET isotope for liposome labeling that allows in vivo tracking of liposomes for up to a week. Desferrioxamine (DFO) is a chelator suitable for ^89^Zr chelation, and with the addition of a weak chelator such as 8-hydroxyquinoline (8-HQ), a high labeling yield of >95% can be achieved, with the resulting radiolabeled liposomes reported to be stable in vitro for up to 48 h and in vivo for up to 15 h [128]. There was a potential splenic radiotoxicity concern reported in a biodistribution study of ^89^Zr-labeled liposomes. High uptake of ^89^Zr-labeled liposomes was recorded in the spleen 48 h post injection, and accumulation continued in the spleen up to 168 h post injection. Later, a decreased volume of the spleen (~2.6-fold difference) was recorded due to the high accumulation of ^89^Zr-labeled liposomes in the spleen. Additionally, results showed that ^89^Zr-labeled liposomes accumulated in the lymph node at a later time point since ^89^Zr has a high affinity for macrophages [128,133]. However, these supporting documents were insufficient for ^89^Zr-labeled liposomes to be approved for use as a diagnostic probe in future clinical settings; hence, further optimization of labeling strategies for liposome labeling with ^89^Zr should be prioritized to overcome the downsides [129]. 

## 4. Conclusions and Future Aspect

This review provides a comprehensive summary of the use of radiolabeled liposomes as nuclear imaging probes. Based on the physicochemical properties and structural characteristics of liposomes, countless efforts have been dedicated toward the development of a suitable labeling method for liposomes with a variety of radionuclides, as well as toward in vivo studies of the use of radiolabeled liposomes as drug carriers and nuclear imaging probes for theranostic applications. The outcomes are still far from meeting expectations, with most of the radioliposomal formulations failing in clinical trials, and none of the liposome-based radiopharmaceuticals have yet entered the current market. This is in spite of the applications of radiolabeled liposomes having shown great potential in nuclear medicine and molecular imaging based on their preclinical data. This review offers perspectives for future research in this regard. Further experiments and strategies should be carried out to assess the efficacy of liposome-based radiopharmaceuticals associated with specific targeting and delivery in various diseases, especially tumor types, which can be achieved by an understanding of tumor heterogeneity, in particular the expression and behavior of tumors in different individuals.

## Figures and Tables

**Table 1:** Comparison of bioimaging modalities.

Imaging Technique	Advantages [23,24]	Disadvantages [23,24]	Imaging Probes [24,25]
PET	γ-ray emission; high sensitivity; unlimited penetration; quantitative imaging	High cost; low spatial resolution (better spatial resolution than SPECT); radiation exposure	Radioisotopes: ^18^F, ^64^Cu, ^124^I, ^68^Ga, ^89^Zr, etc.
SPECT	γ-ray emission; high sensitivity; unlimited penetration depth; radiotherapy	Low spatial resolution; radiation exposure	Radioisotopes: ^99m^Tc, ^111^In, ^67^Ga, ^123^I, ^177^Lu, etc.
CT	X-ray attenuation; high spatial resolution; anatomical imaging	Radiation exposure; limited application	Iodine, barium sulfate, gold particles
MRI	Proton density and relaxation time; high spatial temporal resolution; soft tissue imaging; unlimited penetration	Low sensitivity; long imaging acquisition time; gadolinium health effects [26]	Paramagnetic, superparamagnetic iron oxide nanoparticles, Gd complexes; Gd-based nanoparticles, etc.

**Table 2:** Radiolabeling methods, their advantages, and their disadvantages.

Radiolabeling Method	Radioisotope	Chelator/Other Molecules	Advantages	Disadvantages	Ref.
Passive encapsulation	^99m^Tc	-	Direct incubation during liposome manufacturing	Limited encapsulating capacity, low loading efficiency, low stability, requires freshly prepared liposomes	[42]
	^18^F-fludeoxyglucose (^18^F-FDG)	-			[49,50]
Membrane labeling	^99m^Tc	SnCl_2_ (reducing agent)	High labeling efficiency	Low in vivo stability	[51,52,53]
Remote labeling—ionophore	^111^In, ^67^Ga, ^99m^Tc	nitrilotriacetic acid (NTA), diethylenetriaminepentaacetic acid (DTPA), tetraxetan, 2,2′,2′′,2′′′-(1,4,7,10-tetraazacyclododecane-1,4,7,10-tetrayl)tetraacetic acid (DOTA) Ionophore: A23187, small molecular weight ionophore (oxine)	Labeling efficiency between 60 and 90%	Additional step required for purification procedures	[54,55,56,57,58]
Remote labeling—lipophilic chelator	^99m^Tc	hexamethylpropyleneamine oxime (HMPAO)	Higher labeling efficiency than passive encapsulation	Additional step required for purification procedures	[59]
	^99m^Tc, ^188^Re, ^186^Re	*N*,*N*-bis(2-mercapatoethly)-*N*’,*N*’-diethylenediamine (BMEDA)			[60,61]
	^64^Cu, ^52^Mn	DOTA	-	Poor loading efficiency	[62]
	^64^Cu	Oxine, DOTA	High labeling efficiency and stability; the addition of oxine facilitates the transport of ^64^Cu into liposomes	Synthesis of preformed liposomes that contain oxine	[63,64]
Remote labeling—surface chelator	^89^Zr	Deferoxamine (DFO)	High stability in vitro	Low labeling yield (~44%)	[65]
	^99m^Tc, ^67^Ga	DTPA conjugated to stearylamine	High labeling efficiency	Low in vitro and in vivo stability	[66,67]
	^64^Cu	4-(*N*-hydroxyamino)-2*R*-isobuty-2*S*-(2-thienylthiomethyl)succinyl-l-phenylalanine-*N*-methylamide (BAT)		Additional heating step (>70 °C) required for the incorporation of ^64^Cu into BAT	[68]
	^99m^Tc, ^111^In	*N*-hydroxy succinimidyl hydrazine nicotinate hydrochloride (HYNIC) conjugated to 1,2-Distearoyl-sn-glycero-3-phosphoethanolamine (DSPE)	High stability and labeling efficiency (>95%), rapid labeling procedure without purification required	Limited radionuclide choices	[67,69]
	^99m^Tc-tricarbonyl complex	2-iminothiolane	High labeling efficiency (>90%) and stability	Tedious preparation method required for the synthesis of ^99m^Tc-tricarbonyl (^99m^Tc-(CO_3_^+^))	[54,55,56,57,58,63,64,68,70,71,72,73,74,75,76,77,78,79,80,81,82,83,84]
	^64^Cu, ^52^Mn, ^68^Ga	DOTA conjugated to DSPEIonophore: oxine		Labeling needs to be performed at pH 4 due to the use of ethanol for oxine solubility	[62,63,68]

**Table 3:** Liposomal nuclear imaging probes, their isotopes, applications, and research outcomes.

Liposomal Probe Name	Isotope	Application and Research Outcomes	Ref.
^99m^Tc-labeled liposome	^99m^Tc	In vivo biodistribution study, similar biodistribution in normal tissues of humans and rats	[76]
^99m^Tc-labeled liposome	^99m^Tc	Diagnostic imaging for deep-seated infection	[77]
^99m^Tc-liposomes	^99m^Tc	Diagnostic imaging for focal infection	[78]
^99m^Tc-labeled L1	^99m^Tc	Drug delivery evaluation, potential SPECT imaging probe for arthritis	[79]
^111^In- hosphatidylserine100 (PS100); ^111^In-PS200; ^111^In-labeled phosphatidyl-d-serine100 (DPS100); ^111^In-labeled DPS200; ^111^In- Phosphatidylcholine100 (PC100); ^111^In-PC200	^111^In	Diagnostic imaging for atherosclerotic plaque, high uptake of PS liposomes due to recognition by macrophages	[80]
[^111^In]1% Polyethylene glycols (PEG)2000PS100; [^111^In]5%PEg2000PS100; [^111^In]1%PEG5000PS100; [^111^In]5%PEG5000PS100; [^111^In]PS100; [^111^In]PS200	^111^In	Diagnostic imaging for atherosclerotic plaques, in vivo biodistribution study, slow blood clearance due to PEG modification on the surface of liposomes	[81]
^99m^Tc-labeled liposome	^99m^Tc	Blood pool imaging probe with high in vivo stability	[82]
^99m^Tc-labeled liposomes	^99m^Tc	Diagnostic imaging for staphylococcal abscesses, negatively charged ^99m^Tc-labeled liposomes may be specific for naturally occurring abscesses	[83]
^111^In-labeled liposomes	^111^In	Diagnostic imaging for suspected and unsuspected tumors, in vivo biodistribution study	[84]
^111^In-labeled liposomes (VesCan^®^)	^111^In	Diagnostic imaging for primary tumors or metastases with 70–80% overall sensitivity, in vivo biodistribution study	[100]
^111^In-labeled liposomes	^111^In	Diagnostic imaging for Kaposi sarcoma and lymphoma in AIDS, liposomal encapsulation reduced the toxicity of chemotherapeutics toward healthy tissues	[101]
^111^In-labeled liposomes	^111^In	Diagnostic imaging for malignant glioma, distinct tumor image with no toxicity, low overall brain uptake	[102]
^111^In-labeled VS102 liposomes (VesCan^®^)	^111^In	Diagnostic imaging for carcinomas and in vivo biodistribution	[103]
^99m^Tc- Glutathione (GSH)-liposomes; ^99m^Tc-ammonium sulfate-liposomes; ^186/188^Re-GSH-liposomes; ^186/188^Re-ammonium sulfate-liposomes	^99m^Tc and ^186/188^Re	Diagnostic imaging, radiolabeling technique of preformed liposomes with ^99m^Tc or ^186/188^Re	[104]
^99m^Tc-BMEDA labeled GSH; ^99m^Tc-BMEDA+BT-labeled GSH; ^99m^Tc-BMBuA-labeled GSH; ^99m^Tc-BMBuA+BT-labeled GSH liposomes	^99m^Tc	Radiolabeling method optimization using BMEDA	[105]
^99m^Tc-PEG-HYNIC liposomes	^99m^Tc	SPECT imaging for inflammation, limited to distinguishing between vascularized early adhesions and early abscesses	[106]
^111^In-encapsulated liposomes	^111^In	SPECT imaging probe for the therapeutic efficacy of liposomal anticancer agents	[107]
^67^Cu-small unilamellar vesicles (SUV); ^188^Re-SUV; ^90^Y-SUV; ^131^I-SUV; ^67^Cu- multilamellar vesicles (MLV); ^188^Re-MLV; ^188^Re-MLV; ^67^Cu-G_M1_; ^188^Re-G_M1_; ^90^Y-G_M1_; ^131^I-G_M1_	^67^Cu, ^90^Y, ^188^Re, ^131^I	Liposomal radiopharmaceutical efficacy evaluation	[108]
^188^Re-BMEDA-labeled pegylated liposomes	^188^Re	In vivo biodistribution study, radiolabeling method of liposomes with ^188^Re, theranostic benefit	[109]
^186^Re-(NH_4_)_2_SO_4_ liposomes; ^186^Re-cysteine liposomes	^186^Re	Radiolabeling method for the optimization of ^186^Re, theranostic benefit	[60]
^99m^Tc-BMEDA labeled liposomes with ammonium sulfate, citrate, or GSH	^99m^Tc	Novel radiolabeling method using ^99m^Tc-BMEDA for the labeling of pH/GSH gradient liposomes	[61]
^188^Re-BMEDA-labeled pegylated liposomes	^188^Re	In vivo biodistribution, theranostic vehicle with prolonged half-life due to the novel radiolabeling method using BMEDA for ^188^Re	[110]
^188^Re-BMEDA and ^188^Re-liposome	^188^Re	In vivo biodistribution, theranostic vehicle for lung cancer	[111]
^188^Re-BMEDA-labeled liposome	^188^Re	In vivo biodistribution study, theranostic benefit	[112]
^188^Re-BMEDA-labeled liposome	^188^Re	In vivo biodistribution study, theranostic benefit with long circulation period due to PEG modification	[113]
^188^Re-BMEDA-labeled pegylated liposomal doxorubicin; ^188^Re-liposomes	^188^Re	Therapeutic efficacy study, scintigraphic imaging, in vivo biodistribution, theranostic vehicle	[114]
^188^Re- doxorubicin (DXR)-liposome-BBN; ^188^Re-liposome-BBN	^188^Re	Therapeutic efficacy, scintigraphic imaging, theranostic vehicle	[115]
^188^Re-liposome	^188^Re	Therapeutic efficacy, scintigraphic imaging, theranostic benefit	[116]
Liposomal ^18^[F]-fluorodipalmitin (FDP)	^18^F	Radiolabeling method for the optimization of liposomes, PET imaging probe	[50]
Liposomes modified with ^18^[F]-radiolabeled amphiphilic compounds	^18^F	Radiolabeling method for the optimization of liposomes with ^18^[F]-labeled amphiphilic compounds, PET imaging probe	[117]
^68^Ga-DTPA-PEGylated -liposomes (PLP)	^68^Ga	In vivo biodistribution of radiolabeled liposomes, PET imaging probe	[118]
[^18^F]-fluorinated carboplatin (FCP) encapsulation in [^111^In]-liposomes	^18^F, ^111^In	Dual-tracer PET and SPECT imaging	[119]
^18^F-(*E*)-cyclooct-4-enyl2,5-dioxo1-pyrrolidinylcarbonate(TCO)-liposomes	^18^F	Radiolabeling of liposomes with ^18^F, theranostic vehicle	[120]
^64^Cu-1,4,8,11-tetraazacyclotetradecane-1,4,8,11-tetraacetic acid (TETA) liposomes; ^64^Cu-TETA-PEG2k liposomes; ^64^Cu-CB-1,4,8,11-tetraazabicyclo(6.6.2)hexadecane (TE2A)-PEG2k liposomes	^64^Cu	Radiolabeling method optimization using a maleimide lipid and bifunctional chelator, high stability in vitro and in vivo	[121]
^64^Cu-DPPE-labeled liposomes; ^64^Cu-DSPE-labeled liposomes	^64^Cu	Radiolabeling method and optimization of ^64^Cu for liposomes	[122]
^64^Cu-TATE-liposomes	^64^Cu	Radiolabeling method optimization, in vivo biodistribution study of radiolabeled liposomes, diagnostic imaging for tumors	[123]
^64^Cu-liposomes	^64^Cu	Diagnostic imaging, comparison between ^64^Cu-liposomes and ^18^F-FDG	[124]
^64^Cu-MM-302	^64^Cu	Diagnostic imaging, in vivo biodistribution study of radiolabeled liposomes, theranostic vehicle	[125]
^64^Cu-liposome	^64^Cu	PET imaging probe targeting bone marrow	[126]
^64^Cu-liposome	^64^Cu	Theranostic benefit with improved therapeutic interventions for inflammatory and infectious disease	[127]
^89^Zr-labeled liposomes	^89^Zr	Radiolabeling method optimization of ^89^Zr, in vivo biodistribution study of ^89^Zr-labeled liposomes, visualization of macrophage biology in vivo; the labeling site and the linkers did not alter labeling stability but did alter blood retention time	[128]
[^89^Zr]Zr(oxinate)_4_-liposome complex	^89^Zr	Radiolabeling method optimization of ^89^Zr, PET imaging probe	[129]

## Data Availability

Not applicable.

## Abbreviations

%ID/g—radiolabeled liposome activity, %RCP—percentage of radiochemical purity, ^18F^-FDG—^18^F-fluorodeoxyglucose, 2-IT—2-iminothiolane, 2-HQ—2-hydroxyquinoxaline, 8-HQ—8-hydroxyquinoline, BAT-4-(*N*-hydroxyamino)-2R-isobuty-2S-(2-thienylthiomethyl)succinyl-l-phenylalanine-*N*-methylamide, BHT—butylated hydroxutoluene, BMEDA—*N*,*N*-bis(2-mercapatoethly)-*N*’,*N*’-diethylenediamine, CIA—collagen-induced arthritis, CNS—central nervous system, CT—computerized tomography, CTAB—cetrimonium bromide, DDAB—didodecyldimethylammonium bromide; DFO—deferoxamine, DMPG—1,2-dimyristoyl-sn-glycero-3-phosphocholine, DMPG—dimyristoyl phosphatidylglycerol, DOPS—phospholipid dioleoylphosphatidylserine, DOTAP—dioleoyl-3-trimethylammonium propane, DSPE—1,2-distearoyl-sn-glycero-3-phosphoethanolamine, DTPA—diethylenetriaminepentaacetic acid, DXR—doxorubicin, EDTA—ethylenediaminetetraacetic acid, EPR—enhanced permeability and retention effect, EV—extracellular vesicles, FDA—U.S Food and Drug Administration, GM1—monosialogangioside, GSH—glutathione, GUV—giant unilamellar vesicle, HMPAO—hexamethylpropyleneamine oxime, HPLC—high-performance liquid chromatography, HYNIC—hydrazinonicotinic acid, i.v.—intravenous, keV—kilo electron volts, LUV—large unilamellar vesicle, lyso-PC—1-lysophosphtidylcholine, MLV—multilamellar vesicle, MPS—mononuclear phagocytic system, MRI—magnetic resonance imaging, NTA—nitrilotriacetic acid, PA—phosphatidic acid, PEG—polyethylene glycols, PET—positron emission tomography, PS—phosphatidylserine, PTX—paclitaxel, Ref.—reference, RES—reticuloendothelial system, SD—standard deviation, SEC—size exclusion chromatography, SPECT—single-photon emission computerized tomography, SpHL—pH-sensitive liposome, SUV—small unilamellar vesicle, T_1/2_—half-life, TETA-PDP—(6-(6-(3-(2-pyridyldithio) propanamide)hexanamido)benzyl)-1,4,8,11-tetraazacyclotetradecane—1,4,8,11-tetraacetic acid, TLC—thin-layer chromatography.
